# RNA-Seq Analysis of MCF-7 Breast Cancer Cells Treated with Methyl Gallate Isolated from the Rhizomes of *Nymphaea Odorata* L. Shows Upregulation of Apoptosis, Autophagy, and Unfolded Protein Canonical Pathways

**DOI:** 10.3390/molecules30143022

**Published:** 2025-07-18

**Authors:** Nishikant A. Raut, Pinal N. Kanabar, Mark Maienschein-Cline, Nina S. Los, Zarema Arbieva, Temitope O. Lawal, Shitalben Patel, Gail B. Mahady

**Affiliations:** 1 Department of Pharmacy Practice, University of Illinois at Chicago, Chicago, IL 60612, USA; 2 Research Informatics Core, Research Resources Center, University of Illinois at Chicago, Chicago, IL 60612, USA; 3 Core Genomics Facility, Research Resource Center, University of Illinois at Chicago, Chicago, IL 60612, USA; 4 Department of Pharmacy Practice, College of Pharmacy, World Health Organization Collaborating Centre for Traditional Medicine, University of Illinois at Chicago, Chicago, IL 60612, USA

**Keywords:** *Nymphaea odorata*, apoptosis, autophagy, breast cancer, deep sequencing, unfolded protein response

## Abstract

The effects of a methanol extract of *Nymphaea odorata* (MeNO) rhizomes, its fractions and the active compound (methyl gallate, MeG) were investigated in estrogen receptor-positive (ER+) breast cancer cell lines MCF-7 and T47-D:A18, as well as ER-negative line SKBr3. Cell viability and cytotoxicity were determined using CellTiter-Glo^®^ 2.0 assays at concentrations ranging from 1 to 100 μg/mL. Caspase activity and apoptosis were determined using Caspase-Glo^®^ 3/7, Caspase-Glo^®^ 8, and ApoTox-Glo™ triplex assays, as well as qPCR. Total RNA was isolated from MCF-7 cells treated with MeG. RNA-seq libraries were prepared using a Universal Plus mRNASeq kit, and sequencing was performed on a NovaSeq 6000. MeNO inhibited the growth of MCF-7 cells with an IC_50_ of 14.1 μg/mL, as well as T47-D:A18 (IC_50_ of 25.6 μg/mL) and SKBr3 cells (IC_50_ of 35.5 μg/mL). Bioassay-guided fractionation of MeNO in MCF-7 cells identified the active fraction containing one compound, namely methyl gallate (MeG). MeG had an IC_50_ of 8.6 μg/mL in MCF-7 cells. Transcriptomic analysis of MeG-treated MCF-7 cells showed differential expression of 10,634 genes, with 5643 upregulated and 4991 downregulated (FDR < 0.05). Ingenuity pathway analysis revealed the involvement of 43 canonical pathways, with the top upregulated pathways including apoptosis, autophagy, and the unfolded protein response pathways.

## 1. Introduction

*Nymphaea odorata* Aiton (Nymphaeaceae), also known as the American white waterlily, fragrant waterlily, beaver root, white water lily, and sweet-scented water lily, is a hardy perennial, aquatic plant native to North and South America [1,2,3]. The plant has beautiful, fragrant white or pink flowers; tuberous rhizomes; and large, flat, green floating leaves. Historically, the plant was used by Native Americans and First Nations to treat a wide range of medical conditions, including respiratory and skin infections; sore throats; and gastrointestinal ailments, including diarrhea [2,4]. The entire plant is edible, and the tuberous rhizomes of *N. odorata* are used as a food source by both animals and humans, as they are a good source of protein, carbohydrates, and dietary fibers, as well as phenolic compounds and other phytochemicals [5,6,7].

*Nymphaea odorata* has been traditionally used in some cultures for its potential medicinal properties for the management of numerous disorders. For example, in traditional systems of medicine, extracts of the rhizomes and leaves of *N. odorata* have been used orally for the management of cancer and chronic diarrhea [2,4,7,8]. A tea made from the roots/rhizomes is used for the management of tuberculosis, chronic bronchial complaints, diarrhea, dysentery, gastrointestinal inflammation, vaginal discharge, and mouth ulcers, as well as to stop bleeding [2,4]. A poultice made from the roots is used for the treatment of swelling, boils, skin tumors, and inflamed skin [2,4]. Anecdotally, one case report claimed the complete remission of uterine cancer after treatment with an oral decoction of *N. odorata* and uterine application [4]. In the 19th century, Eli Jones, an American physician and pioneer of American eclectic medicine, utilized *N. odorata* for the treatment of cancer [8]. Numerous *Nymphaea* species are also used in Africa and India for the management of cancer [9,10,11]. While there are some historical and medical reports of the anticancer effects of *N. odorata*, there is little scientific evidence supporting these claims.

In this work, we investigated the anticancer effects of a methanol extract of *N. odorata* (MeNO) rhizomes and its fractions in estrogen receptor-positive (ER+) human breast adenocarcinoma cell lines MCF-7 and T47D:A18, as well as ER-negative line SKBr3. Methyl gallate (MeG) was identified as the active constituent using column chromatography and bioassay-guided fractionation in MCF-7 cells in the most active fraction. Since whole-genome analyses can be used for hypothesis generation with respect to the mechanisms of action and reveal novel gene networks and canonical signaling pathways that are involved in the actions of compounds in cancer cells, we further performed RNA-seq of MeG-treated MCF-7 cells. RNA-Seq, also known as deep sequencing and next-generation sequencing, provides a snapshot of differential gene expression in the transcriptome of cells showing alterations in the quantity and presence of expressed genes (mRNA) in control versus treated cells. Thus, deep sequencing was used to determine the effects of MeG on the transcriptome and identify novel mechanisms of action.

## 2. Results

### 2.1. MeNO Extract Reduced the Viability and Proliferation of Breast Cancer Cells and Induced Apoptosis

**Figure 1:**
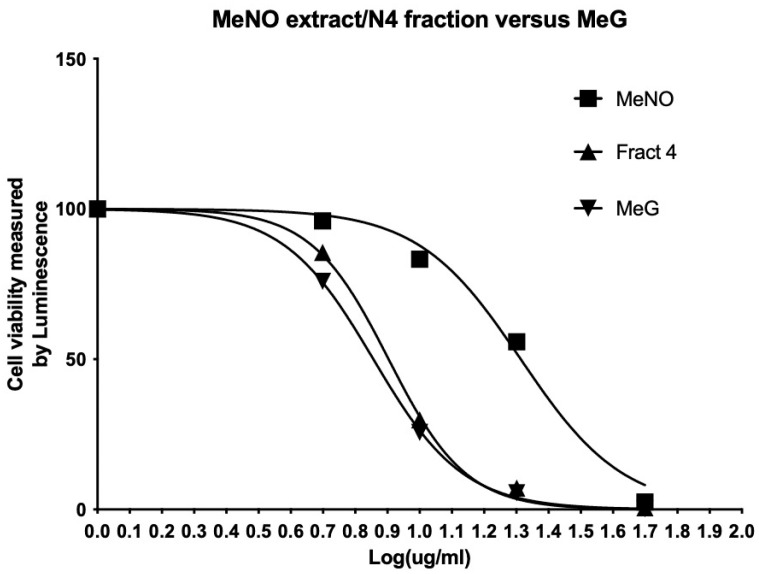
Concentration-dependent inhibition of the viability of MCF-7 cells treated with the methanol extract of *N. odorata* (MeNO), active fraction 4, and methyl gallate (MeG). Median inhibitory concentration (IC_50_) values were calculated using the log (inhibitor) versus the normalized response analysis in GraphPad Prism 10.3. (GraphPad Software, Inc., La Jolla, CA, USA).

MCF-7, T47D:A18, and SK-BR-3 breast cancer cells were grown and maintained as previously described [12,13,14]. Treatment with a methanol extract of *N. odorata* rhizomes (MeNO) significantly reduced the proliferation of MCF-7, T47D:A18, and SK-BR-3 cell lines. The extract inhibited the growth of MCF-7 cells with an IC_50_ of 14.1 μg/mL, T47D:A18 cells with an IC_50_ of 25.6, and SKBr3 cells with an IC_50_ of 35.5 μg/mL (Table 1). The positive control chemotherapeutic drug, 5-fluoruracil (5-FU), had an IC_50_ of 1.37 μg/mL (Table 1). MeNO had a lower IC_50_ in both MCF-7 and T47D:A18 cells, suggesting that the extract may be more effective in hormone receptor-positive breast adenocarcinoma. No significant antiproliferative effects were observed in normal human osteoblasts or rat L6 skeletal myocytes with up to 20 μg/mL of MeNO, and viability remained >95%. Figure 1 shows the time- and concentration-dependent effects of MeNO, an N4 fraction, and methyl gallate (MeG) in MCF-7 cells. The concentration/activity curves and IC_50_ values for N4 and MeG fractions were almost identical, indicating that MeG was the active compound in the N4 fraction. Furthermore, the HPLC of fraction N4 showed the presence of only one compound, a crystalline solid identified using standard UV, IR, NMR, and MS spectrometry, as described in Supplemental Figure S1.

**Figure 2:**
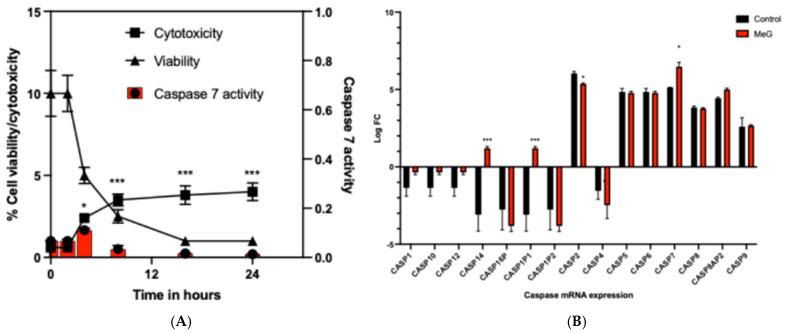
(**A**). Results of treatment of MCF-7 cells with the methanol extract of *N. odorata* in ApoToxGlo™ Triplex Assays for cytotoxicity, viability, and caspase 7 activation. (**B**). Correlative expression of caspase genes in MCF-7 cells treated with the IC_50_ concentration of MeG (data from RNA-seq). Statistics were performed using GraphPad v 10.3 (La Jolla, CA, USA), * *p* < 0.05, *** *p* < 0.001.

**Figure 3:**
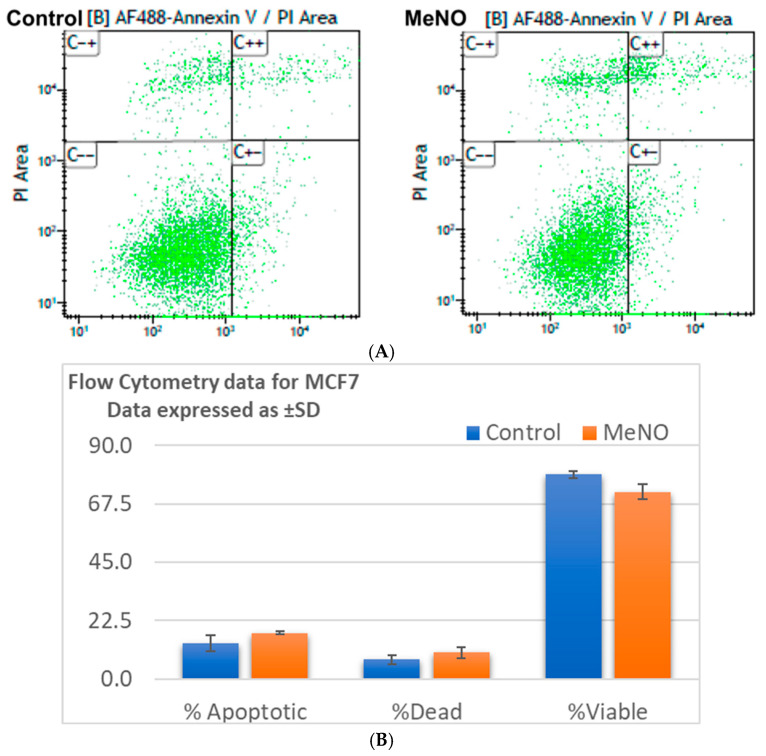

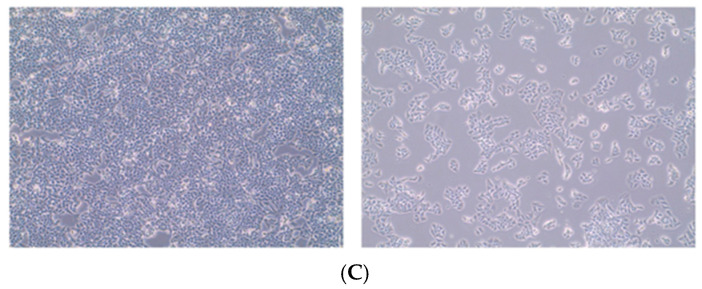
Flow cytometric analysis showed that treatment of MCF-7 breast cancer cells with MeNO induced apoptosis. (**A**). MCF-7 cells were treated with MeNO at the IC_50_ concentration for 12 h, and the cells were harvested and stained with Annexin V and propidium iodide solution. Flow cytometry was performed on a CyAn ADP Analyzer at the UIC Research Resource Center’s Flow Cytometry Core Laboratory. (**B**). MeNO treatment increased the number of MCF-7 cells observed in apoptosis by >16% over 12 h; however, this was not statistically significant as compared with controls using a two-tailed *t*-test (GraphPad v. 10.3, La Jolla, CA, USA). (**C**). MCF-7 cells before and after treatment with MeG at the IC_50_ concentration for 72 h.

To determine if MeNO induced apoptosis in MCF-7 cells, an ApoTox-Glo^®^ Triplex assay kit and flow cytometry were used. In MCF-7 breast cancer cells, MeNO reduced the viability and increased cytotoxicity over 24 h. In the CaspaseGlo^®^ 3/7 functional assay, MeNO increased caspase 7 activity after 4 h of treatment (*p* < 0.05, Figure 2A), indicating induction of apoptosis, but no effects were observed in the CaspaseGlo^®^ 8 functional assay for caspase 8 activity. The increase in caspase 7 activity and caspase expression was correlated with increased cytotoxicity at 8 h (*p* < 0.0001) and decreased cell viability (*p* < 0.01) in MCF-7 cells (Figure 2A,B). Flow cytometry data also showed a trend, suggesting that MeNO induced apoptosis in MCF-7 cells, but this was not statistically significant (Figure 3A,B). Figure 3C shows MCF-7 cells before and after treatment with MeG at the IC_50_ concentration for 72 h.

### 2.2. Methyl Gallate Is Identified as the Active Compound in N. odorata Fraction N4

**Scheme 1:**
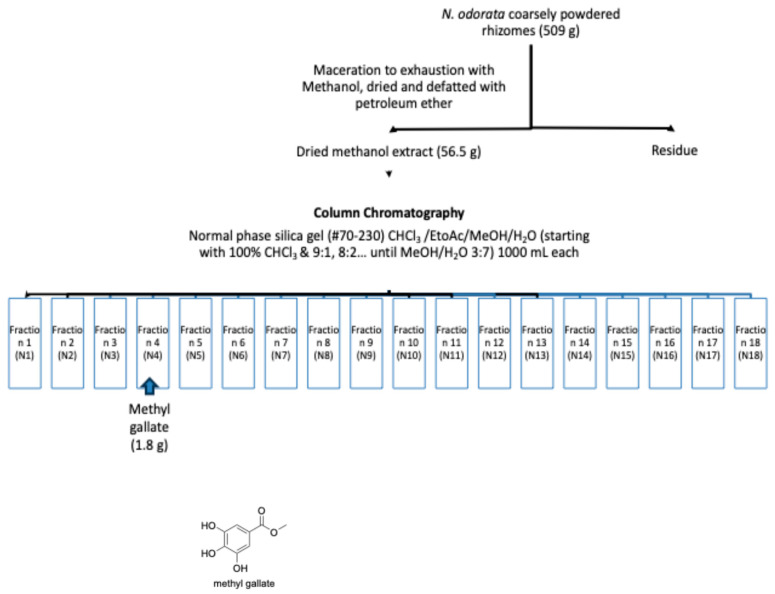
Fractionation scheme for *N. odorata* rhizome extract (MeNO) showing methanol extraction of the powdered rhizome, defatting in petroleum ether, and column chromatography using normal-phase silica gel and a solvent system of CHCl3/EtoAc/MeOH/H2O, resulting in 18 fractions that were tested in the MCF-7 bioassay.

A methanol extract of *N. odorata* rhizomes was fractionated using column chromatography, resulting in 18 fractions, which were tested against MCF-7 cells (Scheme 1). Bioassay-guided fractionation showed that fraction 4 was the most active fraction and reduced MCF-7 cell proliferation in a time-dependent manner (Table 2, Figure 1). The N4 fraction contained only one compound—methyl gallate (MeG)—as a crystalline solid (Supplementary Figure S1). MeG is a known compound and was identified using HPLC, NMR, and mass spectrometry (Supplementary Figure S1). The concentration and time curves for N4 and MeG were almost identical, further confirming that MeG was the active compound in N4 (Figure 1). A bioassay of MeG in MCF-7 breast cancer cells resulted in an IC_50_ of 8.6 μg/mL, while the IC_50_ of the positive control drug, 5-FU, was 1.37 μg/mL (Table 1). RNA-seq analysis of MCF-7 cells treated with MeG (IC_50_) showed a significant increase in the expression of caspases 7 and 14 (FDR < 0.05; Figure 2B).

### 2.3. MeG Alters the Expression of Genes Involved in Apoptosis and Tumor Suppression in MCF-7 Cells

**Figure 4:**
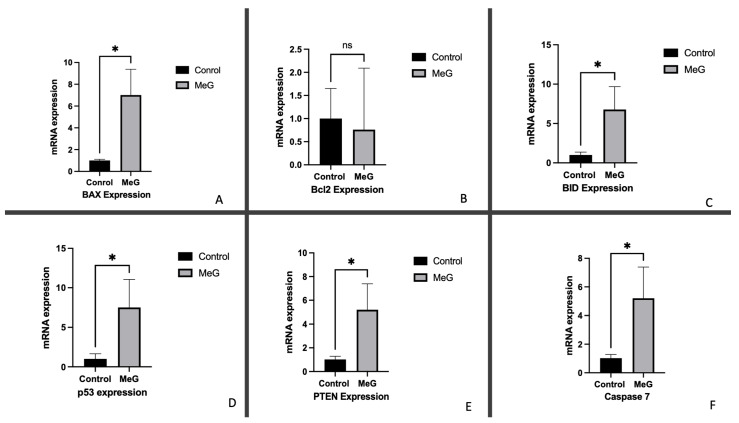
qPCR analysis of Bcl-2 proteins and transcription factors in MeG-treated MCF-7 cells. (**A**) MeG treatment (IC_50_) of MCF-7 cells upregulated *BAX* (pro-apoptotic gene) mRNA expression by >6 fold (*p* < 0.05) over the control, (**B**) downregulated the expression of Bcl-2 mRNA (anti-apoptosis gene) (not significant), (**C**) upregulated *BID* mRNA expression by >6 fold over the control (*p* < 0.05), (**D**) upregulated p53 mRNA expression by >7 fold over the control (*p* < 0.05), (**E**) upregulated *PTEN* mRNA expression by >4 fold over the control (*p* < 0.05), and (**F**) upregulated caspase 7 mRNA expression by ~4.8 fold over the control (*p* < 0.05). Control MCF-7 cells were treated with vehicle solvent (0.01% DMSO) only. Gene expression was quantified using β-actin as the gene with the ^ΔΔ^CT method. Statistics were performed using an unpaired two-tailed *t*-test in GraphPad v10.3 (La Jolla, CA, USA), * *p* < 0.05, ns = not significant.

In MCF-7 cells, caspase 7 activity is known to induce apoptosis by altering the expression of the Bcl-2 protein family. Therefore, we used qPCR to measure the mRNA expression of Bcl-2, Bim, Bid, and Bax, as well as tumor suppressors p53 and PTEN, using qPCR primers as previously described [15,16,17,18] (Figure 4A–F). For the quantitation of gene expression, β-actin was used as the endogenous control gene using the ^ΔΔ^CT method. Treatment of MCF-7 cells with MeG (IC_50_ concentration, 4 h) significantly upregulated the expression of *BAX*, *BID*, *PTEN*, caspase 7, and *Tp53* mRNA (*p* < 0.05) and downregulated *Bcl-2* (not significant). MeG also reduced the expression of *Bcl-2* mRNA, thereby increasing the *Bax/Bcl-2* ratio in favor of apoptosis. qPCR analysis of genes involved in apoptosis and tumor suppression was performed after treatment of MCF-7 cells with MeG (IC_50_).

### 2.4. Transcriptomic Profiling Using RNA-Seq Shows That Methyl Gallate (MeG) Treatment of MCF-7 Breast Cancer Cells Significantly Alters Gene Transcription

Data from mRNA-seq analysis was used to determine the effects of the active compound, i.e., MeG, on the transcriptome of MCF-7 cells, including changes in differential gene expression and their overlap in canonical pathways and molecular networks. MCF-7 breast cancer cells were treated with vehicle solvent (Controls, DMSO 0.01%) or MeG (IC_50_ concentration) in triplicate for 4 h; then, the cells were harvested. Isolated total RNA quality was measured using an Agilent 2100 Bioanalyzer, and the RNA samples had an RNA integrity number score range ≥ 9.6–10. The RNA-seq libraries were prepared and purified, and the library fragment size distribution was between 264 and 294 nt. The libraries were normalized and pooled for a final concentration of 10 nM, then sequenced. Raw data were processed using FastQC for general quality-control metrics, and raw reads were aligned to human reference genome hg38 as described in [16,17,18,19,20,21,22,23]. Differential expression statistics were calculated using EdgeR and the exactTest function, and *p*-values were adjusted for multiple testing using the FDR correction of Benjamini and Hochberg (B&H) [24].

**Figure 5:**
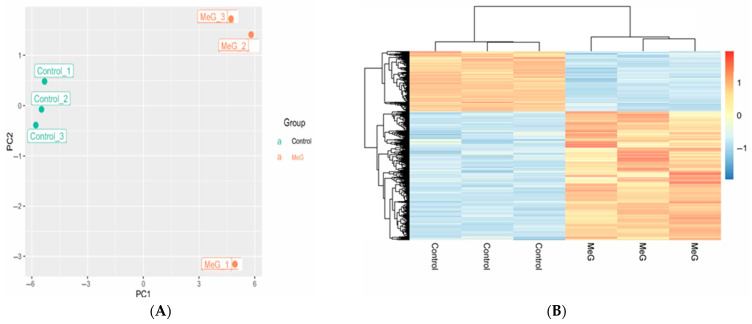
(**A**). Principal component analysis (PCA) of control (MCF-7 cells, *n* = 3, treated with 0.01% DMSO) or MeG (MCF-7 cells, *n* = 3, treated with methyl gallate at the IC_50_ concentration) (three samples each). Control (DMSO 0.01%)—green dots; MeG-treated samples—red dots. (**B**) A heatmap of the top 100 differentially expressed genes (DEGs) with gene ontology across datasets. MCF-7 cells treated with MeG (IC_50_) compared with controls (DMSO 0.05%) using an FDR of q < 0.05 and Log2 FC of <−1 or >1. Each column in the graph depicts the value for one replicate—three for controls and three for the treated cells. The blue and white colors represent downregulated genes, while the yellow and orange colors represent upregulated genes (Z-score log^2^ CPM).

A principal component analysis (PCA; Figure 5A) was generated using the data from the mRNA-seq analysis. The control (0.01% DMSO) and MeG-treated MCF-7 cells were distinct from each other, with the MeG-treated group clustered distantly from controls in the same PCA plot. These data indicate that there was a distinct gene expression pattern for the control versus treatment groups. Transcriptomic analysis of MeG-treated MCF-7 cells showed that of the 26,048 genes analyzed, the expression of 10,634 genes was significantly altered, with 5643 upregulated genes and 4991 downregulated genes (FDR ≤ 0.05). A heatmap of the top 100 differentially expressed genes (DEGs) is presented in Figure 5B, including hierarchical clustering and using a false-discovery rate of ≤0.05 and Log2 FC of ≤−1 or ≥1. As can be clearly seen in the heatmap, there is a significant difference between the patterns of DEGs from the control versus treated cells (Figure 5B), confirming the reliability of the data. Table 3 and Table 4 list the top upregulated and downregulated genes in MCF-7 cells treated with MeG.

### 2.5. Canonical Pathways Impacted in MCF-7 Breast Cancer Cells Treated with MeG

To investigate the overlap of DEGs in MeG-treated MCF-7 cells with specific canonical pathways and molecular networks, Ingenuity^®^ Pathway Analysis software v 24.0 (IPA, Qiagen, Germantown, MD, USA) was used [25]. To measure significance, the ratio of differentially expressed genes from the transcriptomic data was correlated with the total number of reference genes in the canonical pathways in the IPA database using the false-discovery rate (FDR; *q* value) correction of Benjamini and Hochberg (*p* < 0.01) [24].

**Figure 6:**
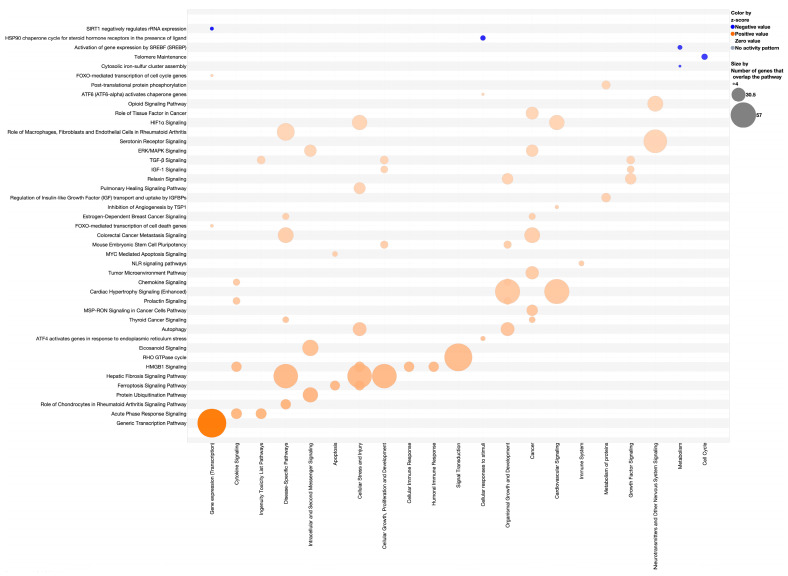
Differential gene expression in cells treated with MeG overlapped with 43 canonical pathways (Log FC of <−1 to >1 and FDR < 0.01). The correlative volcano bubble plot depicts the impacted connected canonical pathways, with the pathway category on the X axis and the name of the canonical pathway depicted on the Y axis. The bubbles in red show upregulation of a pathway, and the blue bubbles represent downregulated pathways. The size of the bubble is representative of the number of genes that overlap in the pathway. The depth of color represents the Z score, with a darker color indicating a higher Z score.

**Figure 7:**
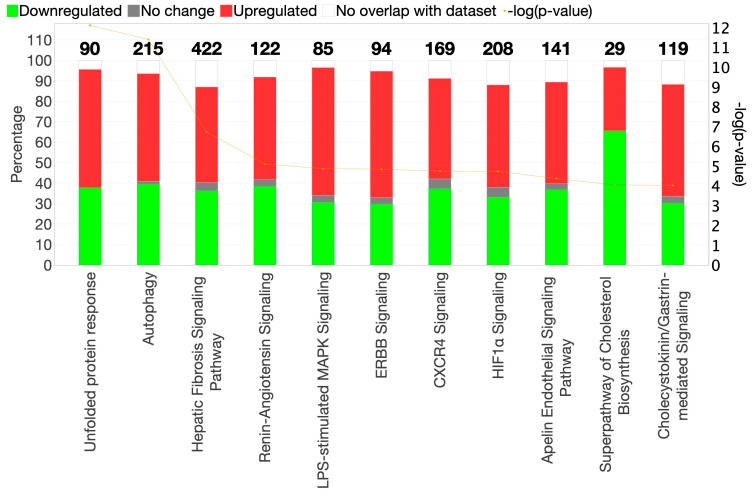
A stacked vertical bar chart showing the top eleven canonical pathways upregulated by MeG in MCF-7 cells (generated in IPA v24.0 using a LogFC of <−1 to >1 and FDR < 0.01. The red represents the percentage of genes upregulated in a particular canonical pathway, while the green represents the percentage of genes downregulated in the pathway. The number of genes in each pathway is given at the top of each bar. The line through the bar chart represents the −log10 (*p* value). A higher −Log (*p* value) indicates a more statistically significant result. A *p*-value of 0.05 becomes −Log10 (0.05) = 1.3, a *p*-value of 0.01 becomes 2, and a *p*-value of 0.001 becomes 3.

**Figure 8:**
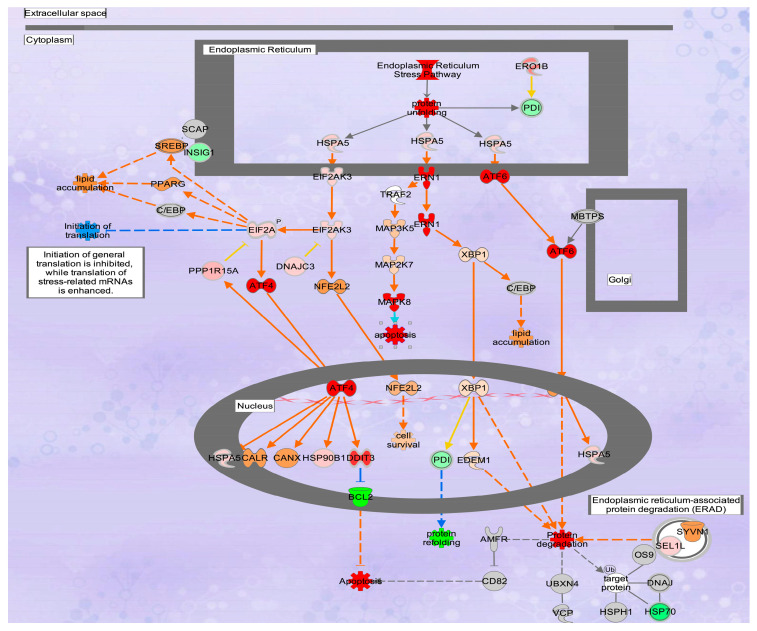
The endoplasmic reticulum stress and Unfolded Protein Response (UPR) canonical pathways were significantly upregulated in MCF-7 breast cancer cells treated with MeG (IC_50_ concentration). Significant experimental upregulation of genes and events are depicted in red/pink, while genes and events shown in in green depict significant downregulation. Genes/pathways in orange or blue represent predicted functions, with orange representing upregulation and blue representing downregulation. Of the 90 genes associated with the UPR, 34 were downregulated and 52 were significantly upregulated in MCF-7 cells treated with MeG, but only genes with a LogFC of ± 3 and a false discovery rate (FDR) of ≤0.01 are shown.

**Figure 9:**
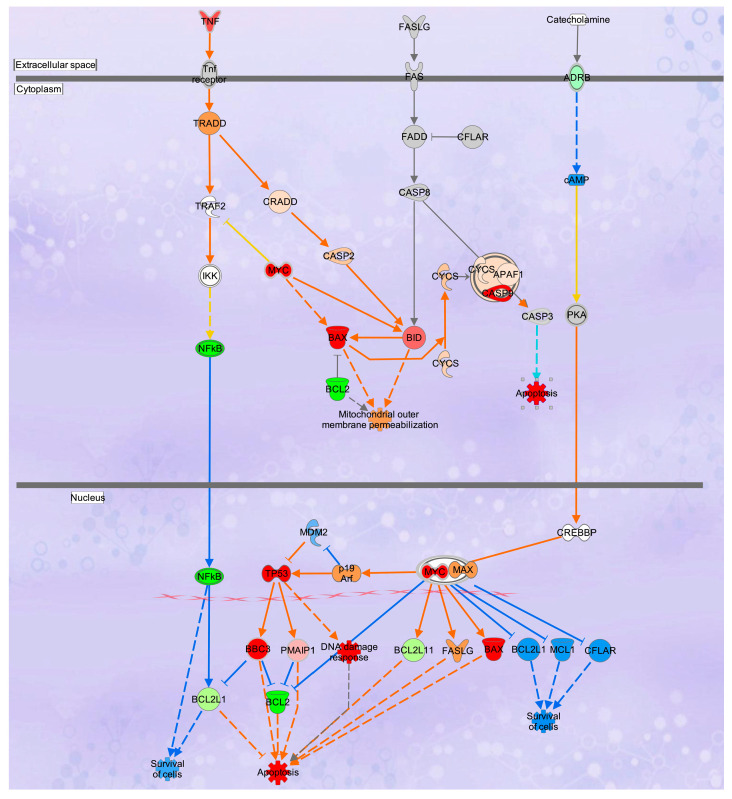
Treatment of MCF-7 cells with methyl gallate (IC_50_ concentration) significantly upregulated the canonical pathway of Myc-mediated apoptosis. Anti-apoptotic genes such as Bcl-2 and Bcl-X were significantly downregulated, while pro-apoptotic genes such as MYC, Bax, Bim, and TP53 were significantly upregulated. Experimentally observed significantly upregulated genes and events are depicted in red/pink, and significantly downregulated genes are depicted in green. Using the prediction function of IPA, genes predicted to be upregulated are presented in orange, while genes predicted to be downregulated are presented in blue. This figure included genes with a LogFC > ±1 and a false-discovery rate ≤0.01. Of the 99 genes in this pathway, 24 were differentially expressed in MCF-7 cells after MeG treatment. Only DEGs with a LogFC > ±1 and FDR < 0.01 are included in this Figure.

**Figure 10:**
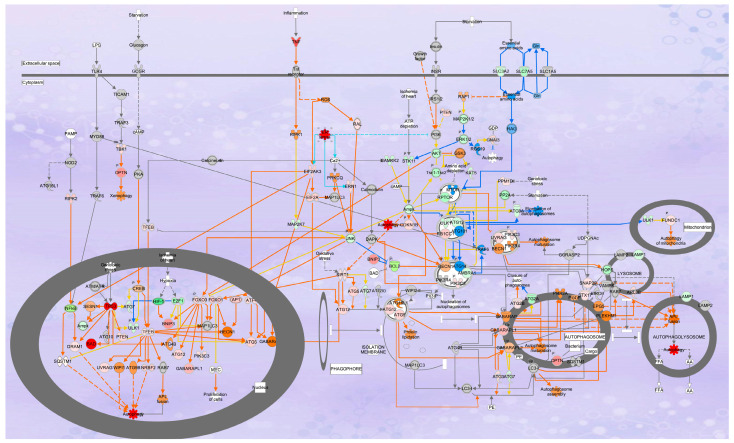
The canonical pathway of autophagy was also significantly upregulated in MCF-7 breast cancer cells treated with MeG (q < 0.05). This pathway contained 215 genes, of which 85 were downregulated and 113 were upregulated in MCF-7 cells treated with MeG. BAD, TNF, and p53 were the most significantly upregulated genes, while Akt, Bcl-2, NF-kB, RPTOR, and MTOR were significantly downregulated. Experimentally observed significantly upregulated genes and events are depicted in red/pink, and significantly downregulated genes are depicted in green. Using the prediction function of IPA, genes predicted to be upregulated are presented in orange, while genes predicted to be downregulated are presented in blue. This figure includes genes with a LogFC > ±1 and a false-discovery rate ≤ 0.01.

Analysis of differential gene enrichment in specific canonical pathways was performed using the ENSEMBL database. Data analyses were based on the ratio of the number of DEGs in the dataset that overlapped with the total number of reference genes in the canonical pathways in the IPA Knowledge databases. The results of these analyses showed that when compared with control MCF-7 cells, differential gene expression in cells treated with MeG overlapped with 43 canonical pathways (LogFC of <−1 to >1 and FDR < 0.01). A bubble plot depicts the impacted connected canonical pathways, with the pathway category on the X axis and the name of the canonical pathway depicted on the Y axis (Figure 6). The bubbles in red show upregulation of a pathway, while the blue bubbles represent downregulated pathways. The size of the bubble is representative of the number of genes that overlap in the specific pathway. The depth of color represents the Z score, with a darker color indicating a higher Z score. The top 11 canonical pathways (Z score > 2.0, q < 0.01) are shown in Figure 7. The unfolded protein response and autophagy canonical pathways were the two most significantly upregulated pathways (Z score > 2.0 and q < 0.01). The most significant cancer-related canonical pathways impacted in MeG-treated MCF-7 cells were the unfolded protein response pathway (Figure 8), apoptosis signaling, myc-mediated apoptosis signaling (Figure 9), and autophagy (Figure 10), indicating that this compound likely has several mechanisms of action in MCF-7 cells. In addition, the expression of NDRG1 (N-myc downstream regulated 1) mRNA, a potent tumor and metastasis suppressor, was significantly upregulated (8.5 fold and q < 0.0001) in MeG-treated MCF-7 cells. Not surprisingly, cancer was the most affected disease state, with gene expression related to cell death, proliferation, survival, and movement significantly impacted. Interestingly, while estrogen-positive breast cancer cells seemed to be more impacted by treatment with MeG, there were no significant changes in the expression of estrogen receptor 1 or 2 and no significant impact on estrogen-dependent cancer signaling in MCF-7 cells treated with MeG (IC_50_).

## 3. Discussion

A methanol extract of white water lily, *N. odorata* (MeNO), reduced the proliferation and viability of MCF-7, T47-D:A18, and SKBr3 breast cancer cells, with the highest activity found in the ER+ MCF-7 and T27D:A18 human breast cancer cell lines (MCF-7), with IC_50_ values of 14.1 μg/mL and 25.6 μg/mL. Analysis flow cytometry and Apotox-Glo^®^ data indicated that MeNO induced apoptosis in MCF-7 cells. While this is the first report of an extract of *N. odorata* suppressing the growth of breast cancer cells, extracts from other *Nymphaea* species have been reported to inhibit the proliferation of MCF-7 cells in vitro [7,26,27,28]. For example, an ethanol extract from *N. nouchali* had significant antioxidant effects and inhibited the proliferation of cultured MCF-7 cells [26]. An ethanol extract of *N. pubescens* flowers weakly inhibited the growth of MCF-7 with an IC_50_ concentration of 91.57 μg/mL [27]. In 2019, Cudalbeanu [28] reported that a methanol extract prepared from *N. alba* leaves and roots inhibited the growth of MCF-7 breast cancer cells. An ethanol extract from the leaves of *N. lotus* was also reported to inhibit the growth of MCF-7 cells with an IC_50_ of 155 μg/mL [10]. However, no active compounds were isolated or identified in these reports. In 2020, Al-Harbi et al. [29] reported that nymphayol, a compound isolated from *N. stellata,* inhibited the proliferation of MCF-7 breast cancer cells with an IC_50_ concentration of 1.4 μM. Our work is the first to report *N. odorata* extracts inducing apoptosis in breast cancer cell lines, with the MCF-7 cell line being the most susceptible, thereby adding to the list of Nymphaea species with anti-breast cancer activities and further suggesting that the *Nymphaea* genus may be an excellent source of new compounds for the prevention and treatment of breast cancer.

Using column chromatography and the bioassay-guided fraction of the MeNO extract in MCF-7 cells, fraction N4 was identified as the most active, and methyl gallate (MeG), a known compound, was isolated and identified from that fraction. Methyl gallate (MeG, C_8_H_8_O_5_) is a naturally occurring phenolic compound with a molecular weight of 184.1461 and is widely found in many plant species, including plants in the *Nymphaea* genus [27,30,31]. The occurrence of methyl gallate has been previously reported in numerous *Nymphaea* species, including *N. alba* [32], *N. ampla* [31], *N. tetragona* [27], *N. pulchella* [31], and *N. stellata* [33]; for the first time in this work, it is reported in a methanol extract of *N. odorata* rhizomes. Previously, only ethyl gallate and gallic acid had been isolated and identified in *N. odorata* [34]. The anticancer effects of MeG have been reported in various cancer and tumor cell lines, such as cervical, hepatocellular, melanoma, and colon cancer cells lines; however, there are very few reports of its effects in breast cancer [30,35,36,37,38,39,40]. In one report, MeG isolated from the kernels of *Mangifera pajang*, a plant from Malaysia, inhibited proliferation and induced apoptosis in MCF-7 breast cancer cells [39]. The IC_50_ for MeG in MCF-7 cells was 81.07 μM [39]. One other investigation of MeG effects in MCF-7 cells reported an IC_50_ of 19.2 μM [40]. In our assays, MeG inhibited the growth and viability of cultured MCF-7 cells with an IC_50_ of 51.9 μM and induced apoptosis via several pathways. The difference in the IC_50_ concentrations between these studies may be due to the purity of the isolated compounds, the type of MCF-7 bioassay used, and the methods used to calculate the median inhibitory concentrations. Previous works have reported that MeG induces cancer cell apoptosis in vitro through the p53 pathway by increasing apoptosis-related protein expression, including that of caspases, and by inhibiting tumor infiltration of CD^4+^CD^25+^ regulatory T cells [35,36,37,38,39]. Our data support these previously published works and show that MeG induces apoptosis in MCF-7 cells via upregulation of caspase 7, Bax, p53, and other apoptosis-related genes. More recently, Choi et al. reported that MeG suppressed mammary tumors in a xenograft mouse model by inducing apoptosis and increasing caspase activity [35]. Although MeG exhibited better activity in ER+ breast cancer cells, transcriptomic analysis showed no significant effects of MeG on the expression of estrogen receptor 1 or 2 and no significant effects on estrogen-dependent cancer signaling pathways.

To investigate other potential mechanisms of action, we used a systems biology approach and performed deep sequencing of MeG-treated MCF-7 cells using RNA-seq. The transcriptome represents all RNA molecules that are present in a population of cells or a single cell at any given time. Changes in the levels of RNA transcripts occur in cells in response to specific conditions or treatments, and these changes can be measured using deep sequencing. Transcriptome sequencing, or RNA-seq, is a next-generation sequencing method used to profile and analyze the changes in RNA transcript levels and provides unbiased information, serving as an extremely precise measurement of transcript levels [41]. Transcriptomic analysis of MCF-7 cells treated with MeG (IC_50_ concentration) showed significant overlap of differentially expressed genes in the canonical pathways of the unfolded protein response (UPR), MYC-mediated apoptosis, and autophagy. Cellular apoptosis (programmed cell death) is a highly regulated process and occurs via two primary pathways—extrinsic or intrinsic—with each involving a cascade of molecular events, including the activation of an initiator caspase (8, 9, or 10), and eventually leading to the activation of the executioner caspases (3 and 7) [41]. For intrinsic (mitochondrial) apoptosis to be initiated, the outer membrane of the mitochondria becomes permeable and releases cell death factors, thereby disrupting mitochondrial function. While the initiation of the extrinsic pathway occurs via activation of the death receptors, a subset of the tumor necrosis factor (TNF) receptor superfamily [41], treatment of MCF-7 cells with MeG significantly upregulated the canonical pathway of extrinsic MYC-mediated apoptosis, including significant upregulation of TNF, Casp7, and 9, as well as MYC mRNA expression (q < 0.05). MYC is a well-known transcription factor, and its upregulation activates Bax and Bak, the primary mediators of MYC-dependent apoptosis [42,43,44,45,46,47]. MYC upregulation in MCF-7 cells by MeG was also associated with significantly downregulated expression (q < 0.05) of Bcl-2 mRNA, an anti-apoptotic protein. Tumor suppressor p53 is also involved in the canonical pathway of MYC-dependent apoptosis [46], and our qPCR and RNA-seq data showed that p53 is significantly upregulated (q < 0.05) in MeG-treated MCF-7 cells. When activated, p53 induces apoptosis by accumulating in the cell nucleus and induces both intrinsic and extrinsic apoptosis pathways [45,46] by upregulating and activating several pro-apoptotic genes, including Bax, as well as downregulating anti-apoptotic proteins, including Bcl-2 [47]. Thus, these data indicate that MeG induces MYC-mediated apoptosis in MCF-7 cells. Interestingly, N-myc downregulated gene 1 (*NDRG1*)*,* a tumor and metastasis suppressor, was significantly (q < 0.0001) upregulated in MeG-treated MCF-7 cells. *NDRG1* is a member of a group of α/β hydrolase proteins that are present in most human tissues, including breast tissue [48]. Downregulation of *NDRG1* mRNA has been associated with increased tumorigenesis in breast cancer [48]. Dose-dependent downregulation of this gene has been associated with 17β-estradiol treatment in ER-dependent cell lines, and upregulation has been reported to be induced by p53 and YAP1 [48].

Further IPA analysis of the transcriptomic data showed a significant overlap of DEG in MeG-treated MCF-7 cells in the canonical pathway of the unfolded protein response (UPR). Approximately 52 of the 90 genes in this pathway were significantly upregulated (q < 0.01). The canonical pathway of UPR maintains cellular protein homeostasis in the endoplasmic reticulum (ENDR) [49,50]. The canonical pathway of UPR is activated when unfolded or misfolded proteins accumulate in the ENDR. The UPR is regulated by the activation of sensors in the ENDR membrane, including activating transcription factors 4 and 6 (ATF4 and 6), inositol-requiring enzyme 1α (IRE1α; ERN1 gene), and pancreatic endoplasmic reticulum kinase (PERK; EIF2AK3 gene) [49]. Upregulation of the UPR results in chronic endoplasmic stress, leading to apoptosis through upregulation of CHOP (DDIT3) expression [49,50]. Analysis of our RNA-seq data showed that gene expression of ATF4 and 6, DDIT3 (CHOP), ERN1, and EIF2A was significantly upregulated (q < 0.01) in MeG-treated MCF-7 cells, indicating the involvement of the canonical pathway of the UPR in the mechanisms of action. Upregulation of DDIT3 (CHOP) activates caspases and inhibits the expression of Bcl-2, inducing apoptosis [50]. Our RNA-seq data support these observations and further suggest that MeG induces MCF-7 cell apoptosis through upregulation of the UPR, likely due to persistent ENDR stress.

Along with apoptosis, activation of the canonical pathway of UPR is also associated with the induction of autophagy [51], another signaling pathway that was significantly upregulated in MeG-treated MCF-7 cells. Autophagy is a process that allows for cellular survival by eliminating or recyclizing components that are unneeded or damaged due to drug treatment, hypoxia, or nutrient deprivation [51]. Increased expression of IRE1 and PERK (EIF2AK3 gene) leads to the inactivation of Bcl-2 and Bcl-XL (two autophagy inhibitory proteins) by phosphorylation, thereby inducing autophagy [50,51]. The activation of PERK also increases autophagy through the induction of both CHOP and ATF4, which results in the inhibition of mTOR [51]. In this work, RNA-seq analysis showed that treatment of MCF-7 cells with MeG significantly (q < 0.01) reduced the expression of mTOR (mammalian target of rapamycin) and RPTOR, its associated regulatory protein. Thus, MeG treatment also induced autophagy in MCF-7 breast cancer cells.

Interestingly, IPA analysis of the RNA-seq data also revealed that MeG significantly (*p* < 0.0001) downregulated the mRNA expression of Tripartite motif-containing protein 28 (TRIM28) and upregulated the expression of numerous associated zinc finger protein (ZFP) mRNAs in MCF-7 cells (Supplemental Figure S2). TRIM28, also known as KRAB-associated protein 1 (KAP1) or transcriptional intermediary factor 1β, regulates gene expression by impacting transcriptional activities and epigenetic regulation of chromatin structure [52,53]. Therefore, TRIM28 plays an important role in multiple cellular processes, including apoptosis, cell growth and differentiation, DNA damage, and maintenance of genomic integrity [52,53,54]. TRIM28 expression is high in MDA-MB-231, MDA-MB-435, T47D, MCF-7, and BT549 breast cancer cell lines and is involved in the development and metastasis of breast cancer [54,55]. Addison et al. [55] showed that TRIM28 levels were increased in cases of metastatic progression of ~40% of invasive breast carcinomas to the lymph nodes. Thus, MeG may also act by reducing the expression of TRIM28 in MCF-7 cells, and further investigations of this are currently underway.

## 4. Materials and Methods

### 4.1. Plant Materials and Extraction

Rhizomes of *Nymphaea odorata* Aiton (Nymphaeaceae; American white waterlily) were collected and identified in Nova Scotia, Canada, by Robert Bruce of Maritime Collections, Nova Scotia, Canada. Herbarium specimens (flowers, leaves, and roots) were deposited at the University of Illinois under collection number RB1102. The rhizomes were cleaned, air-dried, and powdered, and 509 g was extracted in methanol by cold maceration. The extract was dried under reduced pressure using a Buchi Roto-evaporator (Flawil, Switzerland), and the dried extract (MeNO, 1.8 g) was kept at 4 °C until used.

### 4.2. Column Chromatography and Bioassay-Guided Fractionation

The dried methanol extract (56.5 g) was suspended in water (1:1) and defatted with petroleum ether (Scheme 1). The petroleum ether partition was removed and dried. The resultant aqueous/methanol partition was subjected to silica gel column (250 mL) chromatography using normal-phase silica gel (#70-230) and eluted with a solvent system of CHCl3/EtoAc/MeOH/H2O (starting with 100% CHCl3 & 9:1, 8:2… until MeOH/H2O 3:7), using 1000 mL each with successive elution and solvents with an increasing order of polarity. The fractionation resulted in 18 fractions labelled N1–N18 (Scheme 1). Thin layer chromatography was used to identify similar fractions, which were mixed, resulting in a total of 18 fractions. All the fractions were air-dried and stored in a sealed container at 4 °C until use in the bioassay.

The fractions were tested using MCF-7 cells, and IC_50_ values were calculated. Fraction 4 was the most active and showed only one compound (a 1.8 g crystalline solid) by TLC and one peak in HPLC (Supplementary Figure S1A–E). Compound **1** was a crystalline substance and was identified as known compound methyl gallate (IUPAC methyl 3,4,5-trihydroxybenzoate, C_8_H_8_O_5_, Mwt 184.15g) using spectroscopic analyses. Mass spectrum and nuclear magnetic resonance (NMR) for compound **1** were recorded (Supplemental Figure S1D,E). Compound **1** was dissolved in DMSO-d6, and NMR was performed using a 400 MHz Bruker AVIII HD NMR spectrometer (Billerica, MA, USA) equipped with a 5 mm room-temperature SmartProbe™, using TopSpin acquisition and processing software (v3.5).

The fractions obtained from column chromatography were screened for anticancer activity in cultured MCF-7 cells using the same protocol used for the crude extract. The active fractions were subjected to analytical HPLC analysis by a Dionex HPLC system using an Atlantis C18 column (3 µm, 4.6 × 150 mm, Waters, Milford, MA, USA) with a water: methanol gradient of 100, 90:10, 100 and analyzed using Chromeleon Chromatography Software v. 7.3.

### 4.3. Cell Culture Maintenance and Treatment

Human breast cancer cell lines MCF-7 and SKBr3 were purchased from the American Type Culture Collection (Manassas, VA, USA). The T47-D:A18 cell line was cultured as previously described [12,13,14]. MCF-7 cells were grown and sub-cultured as previously described [12,14]. The SKBr3 human breast cancer cell line (estrogen receptor negative, ER^-^) was maintained and sub-cultured as previously described [13]. The cells were sub-cultured onto new media every 5 days at ~80% confluence. The normal cell lines, hFOB human osteoblasts, and L6 rat myocytes were grown and maintained as previously described [56,57].

### 4.4. CellTiter-Glo^®^ Viability Assay

For the viability assays, the cells were plated at a density of 2.5 × 10^4^ cells per 100 μL/well and analyzed using the Promega^®^ CellTiter-Glo^®^ 2.0 (Promega Corporation, Madison, WI, USA) assay as previously described [12,14]. The MeNO, fractions, or MeG were added to the wells at concentrations of 5–100 μg/mL in 0.01% DMSO. The controls included cells treated with 5-Fluorouracil (5-FU) and vehicle control (0.01% DMSO). The plated cells were incubated at 37 °C in 5% CO_2_, and the plate was analyzed as described by the manufacturer using a Synergy HT Plate reader (Biotek, Winooski, VT, USA) and Gen5 1.11 software. The IC_50_ values were calculated using log (inhibitor) versus the normalized response analysis in GraphPad Prism 10.3. For qPCR analysis, reconstituted extracts at the IC_50_ conc. of 14.1 µg/mL were added to experimental wells. Control wells containing culture medium (supplemented with 10% FBS and 1% penicillin/streptomycin) and vehicle control (0.01% DMSO) were store in a humidified incubator at 37 °C in an atmosphere of 5% CO_2_ for 18 h. For mRNA-seq, the cell culture medium was replaced with fresh medium before treatment with MeG at the IC_50_ concentration (8.6 μg/mL), and control cells were treated as described above.

### 4.5. ApoToxGlo™ Triplex Assays (Apoptosis), Caspase-Glo^®^3/7, and Caspase-Glo^®^8

The MCF-7 cell line was cultured as described above, and the assays were performed as instructed by the manufacturer (Promega Corporation, Madison, WI, USA) [12,14]. Luminescence was measured using a Synergy HT Plate reader (Biotek, Winooski, VT, USA) and Gen5 1.11 software.

### 4.6. Flow Cytometry

Flow cytometric measurements were performed using an Alexa Fluor 488 annexin V/dead cell apoptosis kit with Alexa fluor 488 annexin V and PI (Thermo Fisher Scientific, Inc., Waltham, MA, USA) according to the manufacturer’s instructions to determine the occurrence of apoptosis. Briefly, 1 × 10^6^ cells were plated in 6-well plates and allowed to attach overnight. After treatment with MeNO at the IC_50_ for 12 h, the cells were harvested and stained with Annexin V and propidium iodide solution and filtered in 5 mL round-bottom tubes with a cell-strainer cap (BD Falcon, Franklin Lakes, NJ, USA). The apoptosis analysis was performed on a CyAn ADP Analyzer at the UIC Research Resource Center’s Flow Cytometry Core Lab.

### 4.7. RNA Isolation, Purification, and Quantification

Total RNA from treated and control MCF-7 cells was isolated using Trizol (ThermoFisher Scientific, Waltham, MA, USA) and used for both the RNA-Seq and qPCR analyses. RNA quantification and quality control were performed as previously described [12,14]. RNA was used if the RIN numbers were >9.4. A Qubit fluorometer was used to measure the DNA levels (<10%) in the total RNA samples.

### 4.8. Quantitative Polymerase Chain Reaction

Reverse-transcribed RNA was amplified with a Power SYBR Green RNA-to-CT kit (Applied Biosystems, Foster City, CA, USA), using a StepOne Plus Real-Time PCR System (Applied Biosystems, Foster City, CA, USA) as previously described [12,14]. Gene expression was quantified using the ^ΔΔ^CT calculation with B-actin as the endogenous control. Primer sequences, (Table 1) were obtained using previously published work and NIH Primer BLAST v 2.16 [15,16,17,18]. Data was analyzed using Student’s *t*-test for the statistical analyses (GraphPad Software v10.3, La Jolla, CA, USA). Statistical significance was set to *p* < 0.05. Table 5 describes the primer sequences that were used for RT-PCR.

### 4.9. RNA-Seq Library Preparation, Validation, and Quantification

The RNA-seq libraries were prepared with a Universal Plus mRNASeq kit (Tecan, Männedorf, Switzerland) [16,19]. Briefly, the libraries were prepared using 250 ng of total RNA/sample and 15 PCR cycles. The final amplified libraries were purified, and the library fragment size distribution was measured as previously described [16,19]. The concentrations of the final library were determined using qPCR and a KAPA Library Quantification Kit (Roche, Basel, Switzerland; KAPA Biosystems, Wilmington, MA, USA). The final concentration of the library pool was 10 nM and sequenced on a NovaSeq 6000 SP flow cell (Illumina, San Diego, CA, USA) with 2 × 50 nt reads using previously described methods [12,16,19].

### 4.10. Bioinformatics, Statistical Analysis, and Database Annotation

Statistical analysis was performed using GraphPad v 10.3 (La Jolla, CA, USA). Data are presented as the mean plus ± SD. For the qPCR analyses, one-way ANOVA was utilized, followed by Tukey’s multiple comparison test and Dunnett’s multiple comparison test. A value of *p* < 0.05 was considered statistically significant. The median inhibitory concentrations (IC_50_) values were calculated using the log (inhibitor) versus the normalized response analysis in GraphPad Prism 10.3. (GraphPad Software, Inc., La Jolla, CA, USA). For the bioinformatics analyses, generated raw data were analyzed using FastQC as previously described [16,19,20]. FastQC is Java-based software that is freely available (https://www.bioinformatics.babraham.ac.uk/projects/fastqc/ (accessed on 1 May 2025)) and was used for the analysis of FastQ files [20]. STAR and BWA MEM were used to associate the raw reads with human reference genome hg38 [21]. The ENSEMBL database (www.ensembl.org) was used to analyze the biological and molecular functions of DEGs. FeatureCounts was used to quantify ENSEMBL genes [22,23]. Differential expression statistics were computed based on raw expression counts using the exactTest function in edgeR. The Benjamini—Hochberg correction for the false-discovery rate (FDR, q) was used to adjust *p*-values for multiple testing [24].

### 4.11. Ingenuity^®^ Pathway Analysis (IPA)

Ingenuity Pathway Analysis v24.0 (IPA), a software application for analyzing, integrating, and interpreting biological data from RNA-seq, was used to analyze the overlap of differentially expressed genes (DEGs) and canonical pathways [25]. For this work, the “Core Analysis” function was used to interpret RNA-seq data to determine canonical pathways and molecular networks using the predicted protein function in the ENSEMBL database. Differentially expressed genes were filtered using a LogFC of <−1 and >1 or a Z score of 3 and a false discovery (q value) < 0.01. The significance of the canonical pathways and molecular networks were determined using B&H correction [24].

### 4.12. Data Sharing and Availability

The raw data and RNA-seq datasets that support the conclusions of this study were deposited in the National Center for Biotechnology Information Gene Expression Omnibus repository (GEO). These datasets are available using GEO number GSE221019 (https://www.ncbi.nlm.nih.gov/geo (accessed on 15 June 2025)).

## 5. Conclusions

This study provides compelling evidence that *Nymphaea odorata* rhizome extract (MeNO), traditionally used in herbal medicine, possesses significant anti-proliferative effects against breast cancer cell lines MCF-7, T47D:A18, and SkBr3 but no effects on normal cell lines, hFOB human osteoblasts, or L6 rat myocytes. Bioassay-guided fractionation led to the identification of methyl gallate (MeG) as the active compound in fraction N4, which is responsible for the observed cytotoxicity in MCf-7 cells. MeG exhibited potent growth-inhibitory activity and induced apoptosis in MCF-7 cells. Transcriptomic profiling using deep sequencing further revealed that MeG modulates a broad spectrum of gene expression, significantly altering over 10,000 transcripts and activating canonical pathways related to apoptosis, autophagy, and the unfolded protein response. These findings not only validate the ethnopharmacological relevance of *N. odorata* but also highlight MeG as a promising multi-targeted therapeutic candidate for further development in breast cancer treatment. Future studies, including in vivo validations and mechanistic investigations, are warranted to fully elucidate MeG’s therapeutic potential and safety profile.

## Figures and Tables

**Table 1:** Median inhibitory concentrations of *N. odorata* methanol extract, methyl gallate, and 5-fluorouracil in MCF-7, T47D:A18, and SKBr3 breast cancer cells.

Extract/Compound	Breast Cancer Cell Lines and IC_50_ (μg/mL)		
	MCF-7	T47-D:A18	SKBr3
MeNO	14.1	25.6	38.5
MeG	8.6	16.4	19.1
5FU	1.37	2.1	8.5

**Table 2:** Bioassay-guided fractionation of the methanol *N. odorata* extract using column chromatography led to 18 fractions, of which 6 were very active in the MCF-7 bioassay.

Sr. No.	Fraction	% Viability		Sr. No.	Fraction	% Viability	
		100 µg/mL	50 µg/mL			100 µg/mL	50 µg/mL
1	N1	96.23	111.12	10	N12	85.18	95.67
2	**N4**	**1.81**	**1.95**	11	N13	95.12	81.75
3	**N5**	**1.73**	**2.44**	12	N14	13.34	96.99
4	**N6**	**1.97**	**3.11**	13	N15	2.44	11.38
5	**N7**	**4.10**	**4.31**	14	N16	1.97	10.88
6	N8	3.51	32.58	15	**N17**	**0.47**	**24.19**
7	**N9**	**5.43**	**7.14**	16	N18	28.58	98.69
8	N10	2.50	50.24	17	N19	78.55	99.02
9	N11	2.95	101.48	18	N20	63.74	67.94

**Table 3:** Names, ENSEMBL codes, and Log2 Fold Change (FC) of the ten most significantly upregulated genes in MCF-7 breast cancer cells after treatment with MeG (IC_50_ concentration) as compared with the control (DMSO 0.05%) cells using an FDR of q < 0.01 and Log2 FC of <−1 or >1.

Gene Abbreviation	ENSEMBL CODE	Log2FC
KLHDC7B	ENSG00000130487	8.02
INHBE	ENSG00000139269	7.93
NLRP1	ENSG00000091592	7.35
AC0063725	ENSG00000043355	7.24
BEX5	ENSG00000184515	7.22
INFLR1	ENSG00000185436	7.13
SERPINA3	ENST00000555820	6.99
CX3CL1	ENST00000555820	6.79
DIO2	ENST00000555844	6.79
CYBRD1	ENST00000027015	6.72

**Table 4:** Names, ENSENBL codes, and Log2 fold change (FC) of the ten most significantly downregulated genes in MCF-7 breast cancer cells after treatment with MeG (IC_50_ concentration) as compared with the control (DMSO 0.05%) cells using an FDR of q < 0.01 and Log2 FC of <−1 or >1.

Gene Abbreviation	ENSEMBL CODE	Log2FC
LSP1	ENSG00000130592	−6.0
REG4	ENSG00000134193	−5.61
RPL17P15	ENSG00000229326	−5.32
APLN	ENSG00000134817	−4.59
GAB3	ENSG00000160219	−4.37
RAB5C-AS1	ENSG00000267658	−4.17
RP11-90B92	ENSG00000219928	−4.1
MT4	ENSG00000102891	−3.96
IGHA1	ENSG00000211895	−3.94
MEIOB	ENSG00000162039	−3.77

**Table 5:** Forward and backward primers for qPCR.

Forward Primer Sequence (5′ to 3′)	Reverse Primer Sequence (5′ to 3′):
Bcl-2: ′CGCATCAGGAAGGCTAGAGT′	′AGCTTCCAGACATTCGGAGA′
Bax: ′TGCCAGCAAACTGGTGCTCA′	′GCACTCCCGCCACAAAGATG′
β-actin: ′TGACGTGGACATCCGCAAAG′	′CTGGAAGGTGGACAGCGAGG′
p53: ′AAGTCTGTGACTTGCACGTACTCC′	′GTCATGTGCTGTGACTGCTTGRTAG′.
PTEN: ′CCGAAAGGTTTTGCTACCATTCT′	′GTCATGTGCTGTGACTGCTTGRTAG′
Casp 7: ′AGGAGGGACGAACACGTCT′	′CAAAGAAGGTTGCCCCAATCT′
BIM: ′ATGTCTGACTCTGACTCTCG′	′CCTTGTGGCTCTGTCTGTAG′

## Data Availability

The data presented in this study are openly available in [GEO] at [https://www.ncbi.nlm.nih.gov/geo], reference number [GSE221019].

## Supplementary Materials

The following supporting information can be downloaded at: https://www.mdpi.com/article/10.3390/molecules30143022/s1, Figure S1: (A). Fractionation of the methanol extract of N. odorata using column chromatography led to 18 fractions, with fraction N4 having the best activity in MCF-7 cell using bioassay-guided fractionation. Fraction 4 showed only one compound (a crystalline solid, 1.8 g) in HPLC. (B–F). The compound **1** was a crystalline substance and was identified as the known compound methyl-gallate (IUPAC methyl 3,4,5-trihydroxybenzoate, C8H8O5, Mwt 184.15 g) using UV and IR spectroscopic analysis. Mass spectrum and 13C and proton nuclear magnetic resonance (NMR) for compound **1** were recorded in DMSO-d6. NMR was performed using a 400 MHz Bruker AVIII HD NMR spectrometer equipped with a 5 mm room temperature SmartProbe™, using TopSpin acquisition and processing software. Figure S2: RNA-seq analysis of methyl-gallate treated MCF-7 breast cancer cells shows significant (q < 0.01) upregulation of numerous zinc finger proteins and downregulation of Tripartite motif-containing protein 28 (TRIM28) mRNA (TRIM28), a transcription factor involved in the development and metastasis of breast cancer. Experimentally observed significantly upregulated genes and events are depicted in red/pink, and significantly downregulated genes are depicted in green. Using the prediction function of IPA, predicted upregulated genes are presented in orange, while predicted downregulated genes are presented in blue. Of the 99 genes in this pathway, 24 were differentially expressed in MCF-7 cells after MeG treatment. Only DEGs with a LogFC > +1 and FDR < 0.01 were included in this Figure.

## Abbreviations

The following abbreviations are used in this manuscript:

ATF 4/6	Activating transcription factors
GEO	Gene Expression Omnibus repository
IRE1α	Inositol-requiring enzyme 1α
MeG	Methyl gallate
PERK	Pancreatic endoplasmic reticulum kinase
TRIM	Tripartite motif containing 28
UPR	Unfolded protein response

## References

[1] Muhlberg H. (1982). Complete Guide to Water Plants.

[2] Bown D. (1995). Encyclopaedia of Herbs and Their Uses.

[3] Woods K., Hilu K.W., Wiersema J., Borsch T. (2005). Pattern of variation and systematics of *Nymphaea odorata*: I. Evidence from morphology and inter-simple sequence repeats (ISSRs). Syst. Bot..

[4] Foster S., Duke J.A. (1990). A Field Guide to Medicinal Plants. Eastern and Central N. America.

[5] Zhang Z., Elsohly H.N., Cong L.X., Khan S.I., Broedel S.E., Rauli R.E., Cihlar R.L., Burandt C., Walker L.A. (2003). Phenolic compounds from *Nymphaea odorata*. J. Nat. Prod..

[6] Shirly N.H., Chandler R. (1984). Herbal remedies of the maritime Indians: Phytosterols and triterpenes of 67 plants. J. Ethnopharmacol..

[7] Abelti A.L., Teka T.A., Bultosa G. (2023). Review on edible water lilies and lotus: Future food, nutrition and their health benefits. Appl. Food Res..

[8] Grieve A. (1984). Modern Herbal.

[9] Singh M., Jain A.P. (2017). A review on the genus *Nymphaea*: Multi-potential medicinal plant. Asian J. Pharm. Ed. Res..

[10] N’guessan B.B., Asiamah A.D., Arthur N.K., Frimpong-Manso S., Amoateng P., Amponsah S.K., Kukuia K.E., Sarkodie J.A., Opuni K.F., Asiedu-Gyekye I.J. (2021). Ethanolic extract of *Nymphaea lotus* L. (Nymphaeaceae) leaves exhibits in vitro antioxidant, in vivo anti-inflammatory and cytotoxic activities on Jurkat and MCF-7 cancer cell lines. BMC Complement. Med. Ther..

[11] Sowemimo A.A., Fakoya F.A., Awopetu I., Omobuwajo O.R., Adesanya S.A. (2007). Toxicity and mutagenic activity of some selected Nigerian plants. J. Ethnopharmacol..

[12] Kanabar P., Los N., Abrevia Z., Cline M., Patel S., Lawal T.O., Mahady G.B. (2023). Vitamin A, D2 and D3 combinations induced apoptosis and autophagy in breast cancer cells and altered gene expression in the endoplasmic reticulum stress, unfolded protein response and estrogen signaling canonical pathways. FFHD.

[13] Liu J., Burdette J.E., Sun Y., Deng S., Schlecht S.M., Zheng W., Nikolic D., Mahady G.B., van Breemen R.B., Fong H.H. (2004). Isolation of linoleic acid as an estrogenic compound from the fruits of *Vitex agnus-castus* L. (chaste-berry). Phytomedicine.

[14] Lawal T.O., Patel S., Raut N., Mahady G.B. (2021). Extracts of *Anogeissus leiocarpus* and *Dillenia indica* inhibit the growth of MCF-7 breast cancer and COV434 granulosa tumor cells by inducing apoptosis and autophagy. Curr. Bioact. Compd..

[15] NIH Primer BLAST. https://www.ncbi.nlm.nih.gov/tools/primer-blast/index.cgi?GROUP_TARGET=on.

[16] Arbieva Z., Cabada-Aguirre P., Garay Beunrostro K.D., Kanabar P.N., Lawal T.O., Lopez A.M., Los N.S., Maienschein-Cline M., Ostos Mendoza K.C., Patel S.M. (2022). Combination of vitamins A, D2 and D3 reduce tumor-load and alter the expression of miRNAs that regulate genes involved with apoptosis, tumor suppression, and the epithelial-mesenchymal transition in HCT-116 colon cancer cells. Funct. Foods Health Dis..

[17] Liu Y., Bodmer W. (2006). Analysis of P53 mutations and their expression in 56 colorectal cancer cell lines. Proc. Natl. Acad. Sci. USA.

[18] Solomon H., Dinowitz N., Pateras I., Cooks T., Shetzer Y., Molchadsky A., Charni M., Rabani S., Koifman G., Tarcic O. (2018). Mutant p53 gain of function underlies high expression levels of colorectal cancer stem cells markers. Oncogene.

[19] Kanabar P., Los N.S., Lawal T.O., Patel S., Maienshein-Cline M., Arbieva Z., Mahady G.B. (2021). Transcriptomic analysis reveals that combinations of vitamins A, D2 and D3 have synergistic effects in HCT-116 colon cancer cells by altering the expression of genes involved in multiple canonical pathways including apoptosis, regulation of the epithelial mesenchymal transition and immunity. Funct. Foods Health Dis..

[20] Gondane A., Itkonen H.M. (2023). Revealing the history and mystery of RNA-Seq. Curr. Issues Mol. Biol..

[21] Dobin A., Davis C.A., Schlesinger F., Drenkow J., Zaleski C., Jha S., Gingeras T.R. (2013). STAR: Ultrafast universal RNA-seq aligner. Bioinformatics.

[22] Liao Y., Smyth G.K., Shi W. (2014). FeatureCounts: An efficient general-purpose program for assigning sequence reads to genomic features. Bioinformatics.

[23] McCarthy D.J., Chen Y., Smyth G.K. (2012). Differential expression analysis of multifactor RNA-Seq experiments with respect to biological variation. Nucleic Acids Res..

[24] Benjamini Y., Hochberg Y. (1995). Controlling the False Discovery Rate: A Practical and Powerful Approach to Multiple Testing. J. R. Stat. Soc. Ser. B (Methodol.).

[25] Kramer A., Green J., Pollard J., Tugendrich S. (2014). Causal analysis approaches in Ingenuity Pathway Analysis. Bioinformatics.

[26] Ramesh C., Alam A., Murtuja G., Hedayetullah M. (2002). Assessment of in vitro antioxidant and cytotoxic potentials of ethanol extract of *Nymphaea nauchali*. J. Drug Deliv. Ther..

[27] Selvakumari E., Shantha S., Purushoth P.T., Sreenathkumar C. (2012). Antiproliferative activity of ethanolic flower extract from *Nymphaea pubescens* Willd against human cervical and breast carcinoma in vitro. Int. Res. J. Pharm..

[28] Cudalbeanu M., Furdui B., Barbu C., Iancu V., Marques A.V., Dinica R.M. (2019). Antifungal, antitumoral and antioxidant potential of the Danube delta *Nymphaea alba* extracts. Antibiotics.

[29] Al-Harbi L.N., Subash-Babu P., Binobead M.A., Alhussain M.H., AlSedairy S.A., Aloud A.A., Alshatwi A.A. (2020). Potential metabolite nymphayol isolated from water lily (*Nymphaea stellata*) flower inhibits MCF-7 human breast cancer cell growth via upregulation of Cdkn2a, pRb2, p53 and downregulation of PCNA mRNA expressions. Metabolites.

[30] Liang H., Huang Q., Zou L., Wei P., Lu J., Zhang Y. (2023). Methyl gallate: Review of pharmacological activity. Pharmacol. Res..

[31] Marquina S., Bonilla-Barbosa J., Alvarez L. (2005). Comparative phytochemical analysis of four Mexican *Nymphaea* species. Phytochemistry.

[32] Bakr R., Wasfi R., Swilam N., Sallam I.E. (2016). Characterization of the bioactive constituents of *Nymphaea alba* rhizomes and evaluation of anti-biofilm as well as antioxidant and cytotoxic properties. J. Med. Plant Res..

[33] Raja M.K., Sethiya N.K., Mishra S.H. (2010). A comprehensive review on *Nymphaea stellata*: A traditionally used bitter. J. Adv. Pharm. Technol. Res..

[34] Spence S.K. (1997). Bioassay-Directed Isolation of the Allelopathic Constituents of the Aquatic Plant *Nymphaea odorata*. Ph.D. Thesis.

[35] Choi J., Choi J.Y., Jang H., Jang Y., Song J., Kim G., Seol J.W. (2024). Methyl gallate suppresses canine mammary gland tumors by inducing apoptosis and anti-angiogenesis. Anticancer Res..

[36] Huang C.Y., Chang Y.J., Wei P.L., Hung C.S., Wang W. (2021). Methyl gallate, gallic acid-derived compound, inhibits cell proliferation through increasing ROS production and apoptosis in hepatocellular carcinoma cells. PLoS ONE.

[37] Lee H.Y., Lee H.J., Kwon Y.J., Lee J.H., Kim J., Shin M.K., Kim S.H., Bae H. (2010). Methyl gallate exhibits potent antitumor Activities by inhibiting tumor infiltration of CD4+CD25+ regulatory T cells. J. Immunol..

[38] Park J.K., Yoo M.J., Jang H., Park S.Y., Choi J., Seol J.W. (2022). Methyl gallate suppresses tumor development by increasing activation of caspase 3 and disrupting tumor angiogenesis in melanoma. Evid. Based Complement. Altern. Med..

[39] Yazan R., Fadzelly A.B.M., Azlen-Che R., Kartinee K.N., Johnson S., Yuan-Han T., Abdulmannan F., Mohammed S.E. (2022). Methyl gallate isolated from *Mangifera pajang* kernel induces proliferation inhibition and apoptosis in MCF-7 breast cancer cells via oxidative stress. Asian Pac. J. Trop. Biomed..

[40] Kim T.W., Paveen S., Lee Y.H., Lee Y.S. (2014). Comparison of cytotoxic effects of pentagalloyl-glucose, gallic Acid, and its derivatives against human cancer MCF-7 and MDA MB-231 Cells. Bull. Korean Chem. Soc..

[41] Wang Z., Gerstein M., Snyder M. (2009). RNA-Seq: A revolutionary tool for transcriptomics. Nat. Rev. Genet..

[42] Elmore S. (2007). Apoptosis: A review of programmed cell death. Toxicol. Pathol..

[43] Chen H., Liu H., Qing G. (2018). Targeting oncogenic Myc as a strategy for cancer treatment. Signal Transduct. Target. Ther..

[44] Mitchell K.O., Ricci M.S., Miyashita T., Dicker D.T., Jin Z., Reed J.C., El-Deiry W.S. (2000). Bax is a transcriptional target and mediator of c-myc-induced apoptosis. Cancer Res..

[45] Haupt S., Berger M., Goldberg Z., Haupt Y. (2003). Apoptosis: The p53 network. J. Cell Sci..

[46] Wawryk-Gawda E., Chylińska-Wrzos P., Lis-Sochocka M., Chłapek K., Bulak K., Jędrych M., Jodłowska-Jędrych B. (2014). P53 protein in proliferation, repair and apoptosis of cells. Protoplasma.

[47] Hussar P. (2022). Apoptosis regulators Bcl-2 and caspase-3. Encyclopedia.

[48] Saponaro C., Gammaldi N., Cavallo V., Ramírez-Morales M.A., Zito F.A., Sonnessa M., Vari F., Serra I., De Summa S., Giudetti A.M. (2025). Insight into the Regulation of NDRG1 Expression. Int. J. Mol. Sci..

[49] Corazzari M., Gagliardi M., Fimia G.M., Piacentini M. (2017). Endoplasmic reticulum stress, unfolded protein response, and cancer cell fate. Front. Oncol..

[50] Ohoka N., Yoshii S., Hattori T., Onozaki K., Hayashi H. (2005). TRB3, a novel ER stress-inducible gene, is induced via ATF4-CHOP pathway and is involved in cell death. EMBO J..

[51] Verfaillie T., Salazar M., Velasco G., Agostinis P. (2010). Linking ER stress to autophagy: Potential implications for cancer therapy. Int. J. Cell Biol..

[52] Wei C., Cheng J., Zhou B., Zhu L., Khan M.A., He T., Zhou S., He J., Lu X., Chen H. (2016). Tripartite motif containing 28 (TRIM28) promotes breast cancer metastasis by stabilizing TWIST1 protein. Sci. Rep..

[53] Bunch H., Calderwood S.K. (2015). TRIM28 as a novel transcriptional elongation factor. BMC Mol. Biol..

[54] Lyengar S., Farnham P. (2011). KAP1 protein: An enigmatic master regulator of the genome. J. Biol. Chem..

[55] Addison J.B., Koontz C., Fugett J.H., Creighton C.J., Chen D., Farrugia M.K., Padon R.R., Voronkova M.A., McLaughlin S.L., Livengood R.H. (2015). KAP1 promotes proliferation and metastatic progression of breast cancer cells. Cancer Res..

[56] Raut N., Ren Z.T., Lawal T.O., Lee S.M., Mahady G.B. (2021). Cyanidin and Peonidin-3-O-glucoside reduce human osteoblast apoptosis and inhibit RANKL-induced bone loss in transgenic medaka. Phytother. Res..

[57] Mahady G.B., Raut N., Ren Z.T., Lawal T.O., Lee S.M., Salamon I. (2020). Elderberry extracts increase rat L6 myocyte proliferation, reduce apoptosis and act as HDAC1/SIRT1 agonists in vitro. FASEB.

