# Supplementary material for: RNA-Seq Analysis of MCF-7 Breast Cancer Cells Treated with Methyl Gallate Isolated from the Rhizomes of Nymphaea Odorata L. Shows Upregulation of Apoptosis, Autophagy, and Unfolded Protein Canonical Pathways
Source: Molecules. 2025 Jul 18;30(14):3022. doi: 10.3390/molecules30143022 (PMC12298593; doi:10.3390/molecules30143022)
Supplement: Supplementary file 1 [file molecules-30-03022-s001.zip › molecules-3687626-supplementary.pdf]

Supplementary Materials

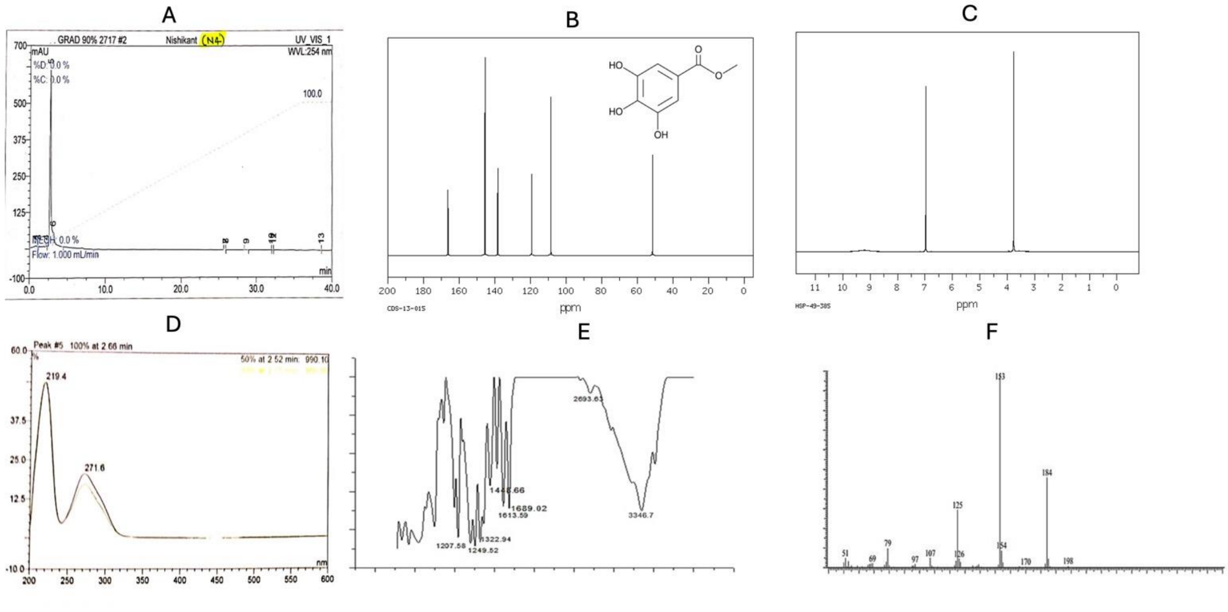

Supplemental Figure S1 A-F. A. Fractionation of the methanol extract of N. odorata using column chromatography led to 18 fractions, with fraction N4 having the best activity in MCF-7 cell using bioassay-guided fractionation. Fraction 4 showed only one compound (a crystalline solid, 1.8 g) in HPLC. **B-F.** The compound 1 was a crystalline substance and was identified as the known compound methyl-gallate (IUPAC methyl 3,4,5-trihydroxybenzoate, C8H8O5, Mwt 184.15g) using UV and IR spectroscopic analysis. Mass spectrum and 13C and proton nuclear magnetic resonance (NMR) for compound 1 were recorded in DMSO-d6. NMR was performed using a 400 MHz Bruker AVIII HD NMR spectrometer equipped with a 5 mm room temperature SmartProbe™, using TopSpin acquisition and processing software.

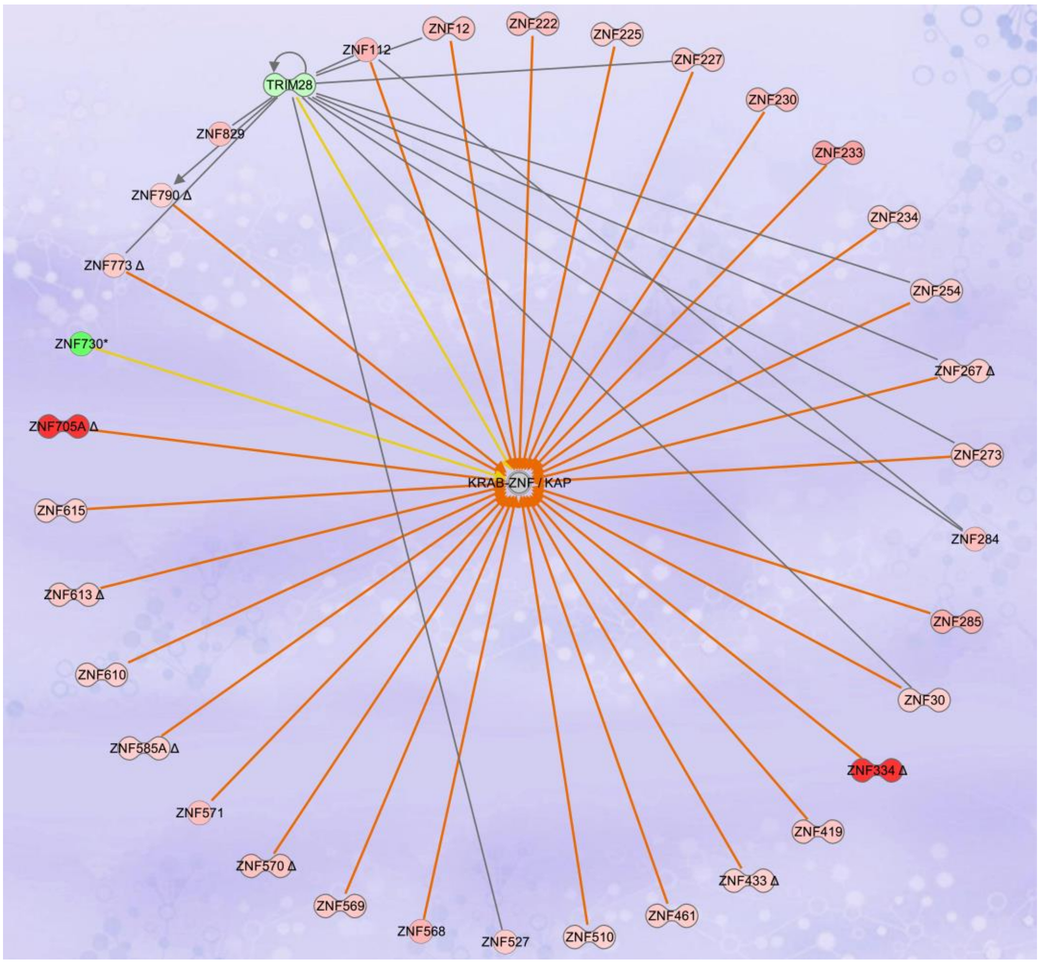

Supplemental Figure S2. RNA-seq analysis of methyl-gallate treated MCF-7 breast cancer cells shows significant ($q < 0.01$) upregulation of numerous zinc finger proteins and downregulation of Tripartite motif-containing protein 28 (TRIM28) mRNA (TRIM28), a transcription factor involved in the development and metastasis of breast cancer. Experimentally observed significantly upregulated genes and events are depicted in red/pink, and significantly downregulated genes are depicted in green. Using the prediction function of IPA, predicted upregulated genes are presented in orange, while predicted downregulated genes are presented in blue. Of the 99 genes in this pathway, 24 were differentially expressed in MCF-7 cells after MeG treatment. Only DEGs with a LogFC > +1 and FDR < 0.01 were included in this Figure.
